# The Role of Zero-Point Vibration and Reactant Attraction in Exothermic Bimolecular Reactions with Submerged Potential Barriers: Theoretical Studies of the R + HBr → RH + Br (R = CH_3_, HO) Systems

**DOI:** 10.1021/acs.jpca.1c05839

**Published:** 2021-09-20

**Authors:** Benjámin Csorba, Péter Szabó, Szabolcs Góger, György Lendvay

**Affiliations:** † Institute of Materials and Environmental Chemistry, Research Centre for Natural Sciences, Magyar tudósok krt. 2., H-1117 Budapest, Hungary; ‡ Center for Natural Sciences, Faculty of Engineering, University of Pannonia, Egyetem u. 10. Veszprém, 8200 Hungary

## Abstract

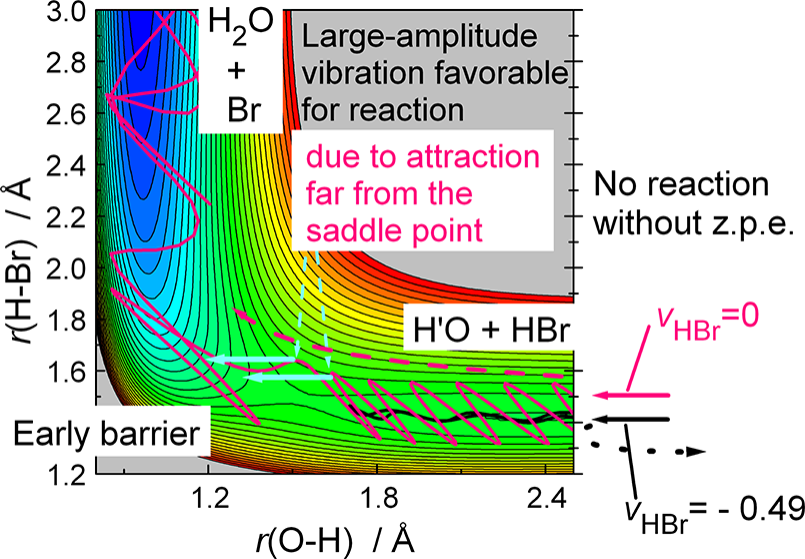

The dynamics of the reactions CH_3_ + HBr → CH_4_ + Br and HO + HBr → H_2_O + Br have been studied using the quasiclassical trajectory method to explore the interplay of the vibrational excitation of the breaking bond and the potential energy surface characterized by a prereaction van der Waals well and a submerged barrier to reaction. The attraction between the reactants is favorable for the reaction, because it brings together the reactants without any energy investment. The reaction can be thought to be controlled by capture. The trajectory calculations indeed provide excitation functions typical to capture: the reaction cross sections diverge when the collision energy is reduced toward zero. Excitation of reactant vibration accelerates both reactions. The barrier on the potential surface is so early that the coupling between the degrees of freedom at the saddle point geometry is negligible. However, the trajectory calculations show that when the breaking bond is stretched at the time of the encounter, an attractive force arises, as if the radical approached a HBr molecule whose bond is partially broken. As a result, the dynamics of the reaction are controlled more by the temporary “dynamical”, vibrationally induced than by the “static” van der Waals attraction even when the reactants are in vibrational ground state. The cross sections are shown to drop to very small values when the amplitude of the breaking bond’s vibration is artificially reduced, which provides an estimate of the reactivity due to the “static” attraction. Without zero-point vibration these reactions would be very slow, which is a manifestation of a unique quantum effect. Reactions where the reactivity is determined by dynamical factors such as the vibrationally enhanced attraction are found to be beyond the range of applicability of Polanyi’s rules.

## Introduction

**Figure 1:**
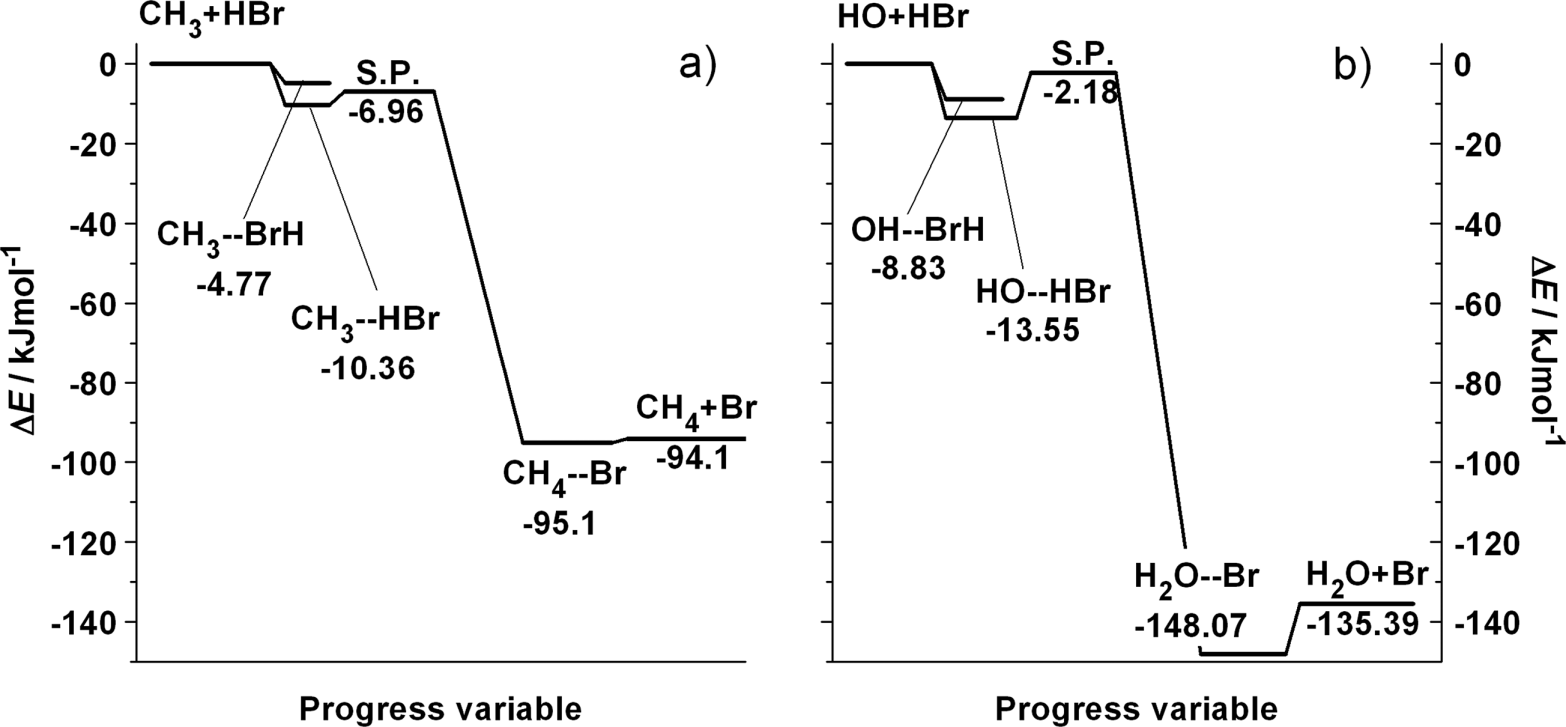
Potential energy profiles of the (a) CH_3_ + HBr (R1) and (b) HO + HBr (R2) reactions. The classical energy levels of the stationary points characterizing the Czakó-Góger-Szabó-Lendvay^20^ potential energy surface are given in kJ/mol. S.P. denotes the saddle point.

Hydrogen abstraction reactions by halogen atoms and by OH radicals from small molecules are prototypes of bimolecular atom-transfer reactions. As they lend themselves to detailed experimental and theoretical reaction dynamical studies, they also serve as testing grounds of theories on the general rules of reaction dynamics. A characteristic feature of these reactions is that on their potential energy surfaces (PESs) shallow potential wells appear due to the attractive long-range forces between the two reactant as well as the two product particles.^1^ When both reactants are polar, the depth of the well corresponding to the prereaction complex generally exceeds 10 kJ/mol,^2−5^ especially when well-defined hydrogen bonds can be formed.^6^ When only one of the reactants is polar, then the well is significantly less deep,^7−9^ and the attractive forces acting at a long-range are rather weak. The presence of the potential well can influence the dynamics of the reaction, as demonstrated for the reactions of F atoms with H_2_O^10^ and with methane,^11^ but little is known about the general rules. In the class of reactions of hydrogen halides with alkyl and other radicals, those involving Br atoms attracted attention because of their importance in atmospheric chemistry^12,13^ and flame retardation.^14^ The experimental studies of H atom-abstraction reactions from HBr were instrumental in the determination of heats of formation of the radicals.^15−18^ For the reactions of HBr with methyl radicals
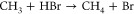
R1and with OH radicals
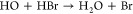
R2the experiments reported negative activation energy.^15,18,19^ The curiosity of these reactions is that, because a weak H–Br bond is broken and a much stronger C–H or O–H bond is formed, they are significantly exothermic. The potential barrier to reaction is early and low, in agreement with the expectation for exoergic reactions, based on the Bell–Evans–Polányi^21,22^ principle. According to high-level ab initio calculations, the attraction between the reactants modifies this picture: the geometry of the reactants at the saddle point corresponding to H-abstraction on the PES becomes so reactant-like that the breaking H–Br bond is hardly longer than the equilibrium distance (1.487 vs 1.413 Å for both reactions), and the forming bond is as long as 1.7 and 1.551 Å for reactions R1(9,20) and R2, respectively.^23^ Moreover, the potential barrier submerges below the reactant level, as shown in Figure 1, which means that the primary factor determining the dynamics, especially at low collision energies, can be assumed to be the attractive potential leading to the prereaction van der Waals well. In general, the dynamical consequence of the long-range attraction is that the reaction cross sections are very large at low collision energy and decrease quickly when the latter increases. The phenomenon when the reactants attract each other, and there is no barrier to the reaction so that the majority of collisions lead to reaction, is called capture.^24^ Capture-type behavior is characterized by excitation functions diverging with decreasing collision energy. The rate coefficients corresponding to this kind of excitation function are characterized by negative activation energy, as are those observed for both reactions R1 and R2. For reaction R1, diverging cross sections at decreasing collision energy have been observed in recent quasiclassical trajectory and reduced-dimensional quantum dynamical calculations.^25^ For reaction R2, de Oliveira et al.^23^ reported excitation functions that seem to show extremely fast divergence of the reaction cross sections when the collision energy decreases, which was supported by the quantum dynamical calculations of D. Wang et al.^26^ The cross sections observed in molecular beam experiments by Kasai and co-workers^27,28^ also increase with decreasing collision energy. For reactions with an early barrier, such as reactions R1 and R2, Polanyi rules of reaction dynamics^29^ predict that vibrational excitation of the reactants promotes the reaction less efficiently than an equal amount of translational energy. The rules do not exclude that reactant vibration (in fact, that of the breaking bond) enhances the reactivity; the focus is on the relative efficiency with respect to translation. These rules apply to reactions with positive potential barriers, and not much is known about how the presence of the prereaction potential well interferes with them. For reaction R1, the currently available information on how the vibrational excitation of the HBr molecule, i.e., the breaking bond, influences the reaction cross sections comes from the theoretical study by Y. Wang et al.,^30^ who performed reduced-dimensional quantum scattering calculations on reaction R1 on an analytical potential energy surface.^31^ On the PES they used, the barrier to reaction is positive, and accordingly, the excitation functions they obtained for the reaction of HBr in the vibrational ground state correspond to what is referred to as activated behavior: the cross sections are zero below a threshold and rise slowly above it. However, for HBr excited by one vibrational quantum, the shape of the excitation function is qualitatively different: the cross sections are very large at low collision energy and decrease quickly with rising collision energy, i.e., they display capture-type behavior. The observation of this kind of excitation function seems to contradict the presence of the potential barrier on the PES.

Useful information also comes from studies of reactions related to R2. Guo and co-workers^32,33^ studied the effect of reactant vibrational excitation of two reactions of the HO + HX family, with X = F and with X = Cl. The reaction of HF is endothermic with a high, late barrier, while that of HCl is exothermic with a low, early barrier. In both reactions, when the HX reactant is in vibrational ground state, the cross sections are zero below a threshold associated with the potential barrier, in agreement with the expected activated behavior. However, when the HX reactant is vibrationally excited, the change of the excitation functions differs for the two reactions. For the reaction of HF, with respect to the ground-state reaction, the threshold energy is reduced by approximately the energy of the vibrational quantum and the cross sections grow an order of magnitude faster. On the other hand, for the reaction of HCl, reactant vibrational excitation changes the character of the excitation function from activated to capture-type.

That vibrational excitation can switch the character of the excitation function is not unknown in the literature. Smith and co-workers^34^ observed extreme, 16 orders of magnitude speed-up for the H + H_2_O → H_2_ + OH reaction when the local O–H stretch mode of water was excited by three or four vibrational quanta. The reaction is endothermic and has a late barrier, so in light of Polanyi’s rule, the fact that the rate increases by vibrational excitation of the reactant is not surprising, but the magnitude is. The explanation, the appearance of capture-type excitation functions at vibrational excitation by more than 2 quanta, was provided by the related theoretical studies.^35−38^ The phenomenon was also observed for the very endothermic H + HF^37,39^ and for the almost thermoneutral H + HCl^40^ hydrogen-abstraction reactions. According to quasiclassical trajectory calculations, the switch of the excitation function from activated to capture-type was found to take place as soon as the energy content of the HX vibration exceeded the potential barrier, which occurred at excitation by about 2.5 quanta for HF and below 2 quanta for HCl. The reason for the drastic change of the excitation functions was traced back to the shape of the potential energy surface. When the breaking bond is highly excited, the amplitude of the X–H bond length oscillation is very large. When the vibration is at the outer turning point (OTP), the H atom is so far from the X atom that the bond will behave as partially broken, which makes it easy for the approaching reactant to abstract the H atom. In terms of the PES, this is manifested when one calculates the potential energy as a function of the length of the forming bond at fixed values of the breaking bond length: As shown in Figure S1 for the reaction of HF with H, at equilibrium H–X distance the potential energy increases monotonously as the reactants approach each other. When the length of the breaking H–X bond is increased, the rate of the potential energy increase slows down, and above a certain H–X distance, the curves become attractive (see Figure S1b). When at the time of the encounter of the approaching reactants the breaking bond is near the OTP of its vibration, this attraction pulls the reactants together.

Reactant vibration with large amplitude can induce unexpected features to the dynamics of reactions in which the potential barrier to reaction is submerged as a reef and is located very close in the configuration space to the minimum of a pre-reaction well. In such cases, the reacting system has enough energy to glide over the potential barrier. If the reactants are captured in the well for a long enough time, and energy is completely redistributed, then the outcome of the encounter depends only on statistical factors. In such cases, only the magnitude of the total energy made available to the reactants counts; its source does not (subject to angular momentum conservation). However, considering that the coupling between the degrees of freedom in a van der Waals complex is weak, one cannot expect complete energy redistribution. Thus, there is a good chance that the effect of vibrational and translational energy on the reactivity is different.

We intend to explore the influence of vibrational excitation of the reactants on the dynamics of reactions R1 and R2 and find an explanation to the unexpectedly fast divergence of the cross sections at low collision energy. In the rest of the manuscript, first we briefly summarize the methodology, and then present the excitation functions and opacity functions at different conditions. This will be followed by an analysis of the potential energy surfaces and the role of the amplitude of the initial reactant vibration, with special attention to its magnitude in the vibrational ground state. We will vary the amplitude with various methods, changing the vibrational quantum number beyond that dictated by semiclassical quantization. Finally, we briefly discuss the connection between the observed vibrational effect and Polanyi’s rules.

## Methods

The dynamics of reactions R1 and R2 have been studied by standard quasiclassical trajectory (QCT) calculations.^41,42^ Initial conditions were generated by the Raff–Porter–Miller scheme^43^ for the diatomic reactants and by normal mode sampling for the CH_3_ radical.^44−46^ For testing the possible temporal evolution of the internal state of CH_3_ during the initial free flight, we utilized the observation^47,48^ that if sets of trajectories are started at various initial reactant separations, thus allowing different initial flight time, the reaction cross sections oscillate or systematically increase or decrease if plotted against the initial distance. The details of the test have been described in ref (25). Briefly, sets of 16 000 trajectories were integrated starting from different center-of-mass reactant distances in the range of covering a 12 to 32 Å at two collision energies. This range of initial distances corresponds to a flight time range at least 50 periods of C–H vibration. The reaction cross sections were found to fluctuate within an 8% range of the average, without any tendency.

The maximum impact parameter was varied according to the collision energy. The production calculations were performed with impact parameters that were large enough to ensure that no reactive collisions occur in the 0.5 Å wide outer ring of the target. At low collision energies, maximum impact parameters as large as 14 Å were needed to get converged reaction probabilities and cross sections, while at large relative velocities, 4.5 Å is satisfactory. These values are similar to those observed for the H + H_2_O(*v*_stretch_ = 4) reaction.^36−38^ Trajectories were integrated with the velocity–Verlet and the Runge–Kutta–Gill method for reactions R1 and R2, respectively, with time steps 0.1 or 0.07 fs, ensuring energy conservation to better than 0.05 kJ/mol. All reactive collisions were included in the cross-section calculations, without any weighting. This method resulted in good agreement with excitation functions provided by reduced-dimensional quantum scattering calculations^25^ and with the experimental thermal rate coefficients^23^ for reaction R1. For the calculation of excitation functions, 124 000 trajectories were integrated at each collision energy and vibrational state. Opacity functions were determined by running 50 000 trajectories at each impact parameter The statistical errors of the quantities obtained with the Monte Carlo QCT method were calculated using the standard prescription,^41,42^ and in most cases are as small as the size of symbols shown in the plots. The calculations were performed using an extensively modified version^40,49−51^ of the VENUS code.^52^ The potential energy surface developed earlier,^9,23^ referred to as CGSL PES was used for the simulations of reaction R1, while the trajectory calculations for reaction R2 were performed using the PES of de Oliveira et al.^23^

## Results

### Cross Sections

**Figure 2:**
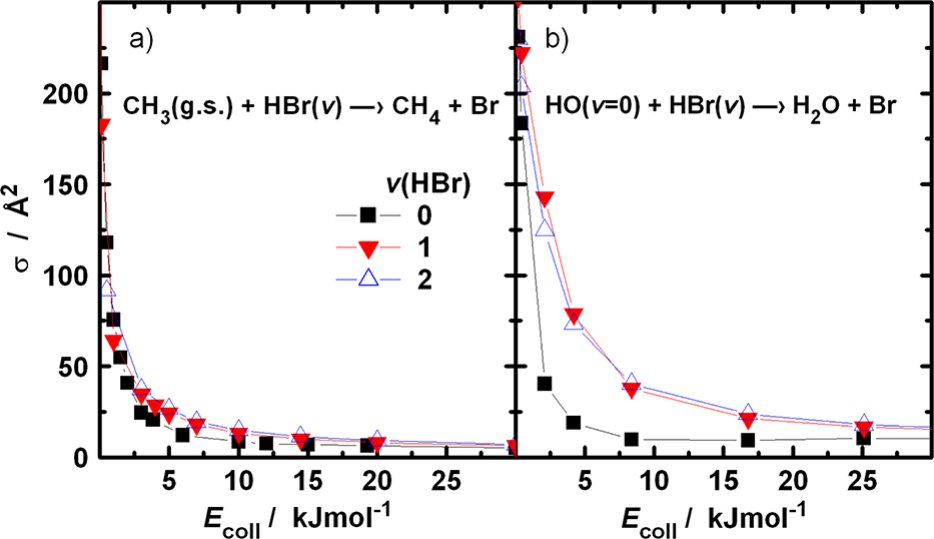
Excitation functions of the (a) CH_3_ + HBr(*v*) and (b) HO + HBr(*v*) reaction for *v* = 0, 1, and 2. The methyl and HO radicals are in their vib-rotational ground state. The error bars calculated with the standard Monte Carlo formula are smaller than the symbol size.

The excitation functions for reactions R1 and R2 are plotted in Figure 2 for the ground and first and second excited states of the HBr reactant. The reactive cross sections diverge quickly as the collision energy decreases, which is the origin of the negative activation energy observed both experimentally^15−18^ and in simulations.^25^ If the submerged barrier were not present, the divergence could be considered to be the natural consequence of the long-range attraction between the reactants. The appearance of divergent cross sections indicates that the decisive factor—at least at low collision energies—is the attractive potential, and the potential barrier plays a secondary role. The magnitude of the reactivity enhancement with decreasing collision energy, however, seems to be rather large if one considers that the PES on the reactant side is flat, and the small long-range attraction leads to a shallow potential well instead of the many kJ/mol deep minima in ion–molecule and radical–radical reactions.

Vibrational excitation of HBr by one quantum is favorable for both reactions. The shapes of the excitation functions remain similar to those for the ground state, but they run above the latter. The enhancement of reactivity is not surprising, since the bond to be broken is excited. The enhancement factor, the ratio of the cross sections for *v*(HBr) = 1 to those at *v*(HBr) = 0 at identical collision energies (shown in Figure S2), is the largest at around *E*_coll_ = 10 kJ/mol, where the cross sections for *v*(HBr) = 1 are larger than for *v*(HBr) = 0 by about a factor of 1.6 and 4 for reactions R1 and R2, respectively, and decreases when the collision energy increases or decreases. When HBr is excited by a second vibrational quantum, the additional enhancement of the cross sections is significantly smaller than that induced by the first. The *v*(HBr) = 2 to *v*(HBr) = 1 enhancement factor is not larger than 1.16 and 1.11 for reactions R1 and R2, respectively, at any collision energy. At low collision energy, the three excitation functions converge. This indicates that the efficiency of reactant vibrational excitation does not increase without limits with the vibrational energy; instead, a saturation effect can be observed.

### Opacity Functions

**Figure 3:**
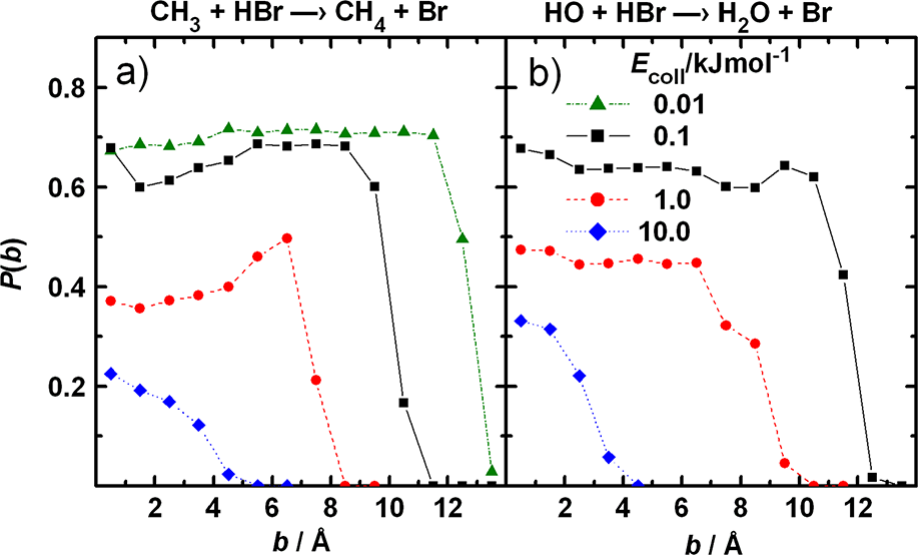
Opacity functions of the (a) CH_3_ + HBr and (b) HO + HBr reaction at various collision energies. All reactants are in vib-rotational ground state.

In Figure 3, the opacity functions for reactions R1 and R2 are plotted at selected collision energies. The change of the shape is typical for reactions with attractive potential. At very low collision energy, the reaction probabilities are large, around 0.7 in the entire impact parameter range extending, for example, up to 12 Å at *E*_coll_ = 0.01 kJ/mol for reaction R1, where they suddenly drop. This limit marks the location of the (orientation averaged) centrifugal barrier. Capture, in fact, does not guarantee that reaction occurs, even though the energetic requirements are fulfilled. With increasing collision energy, the opacity functions shrink, and for reaction R1 a maximum appears near the high-impact-parameter end of the curves, which disappear when the collision energy is above about 5 kJ/mol.

### Individual Trajectories

**Figure 4:**
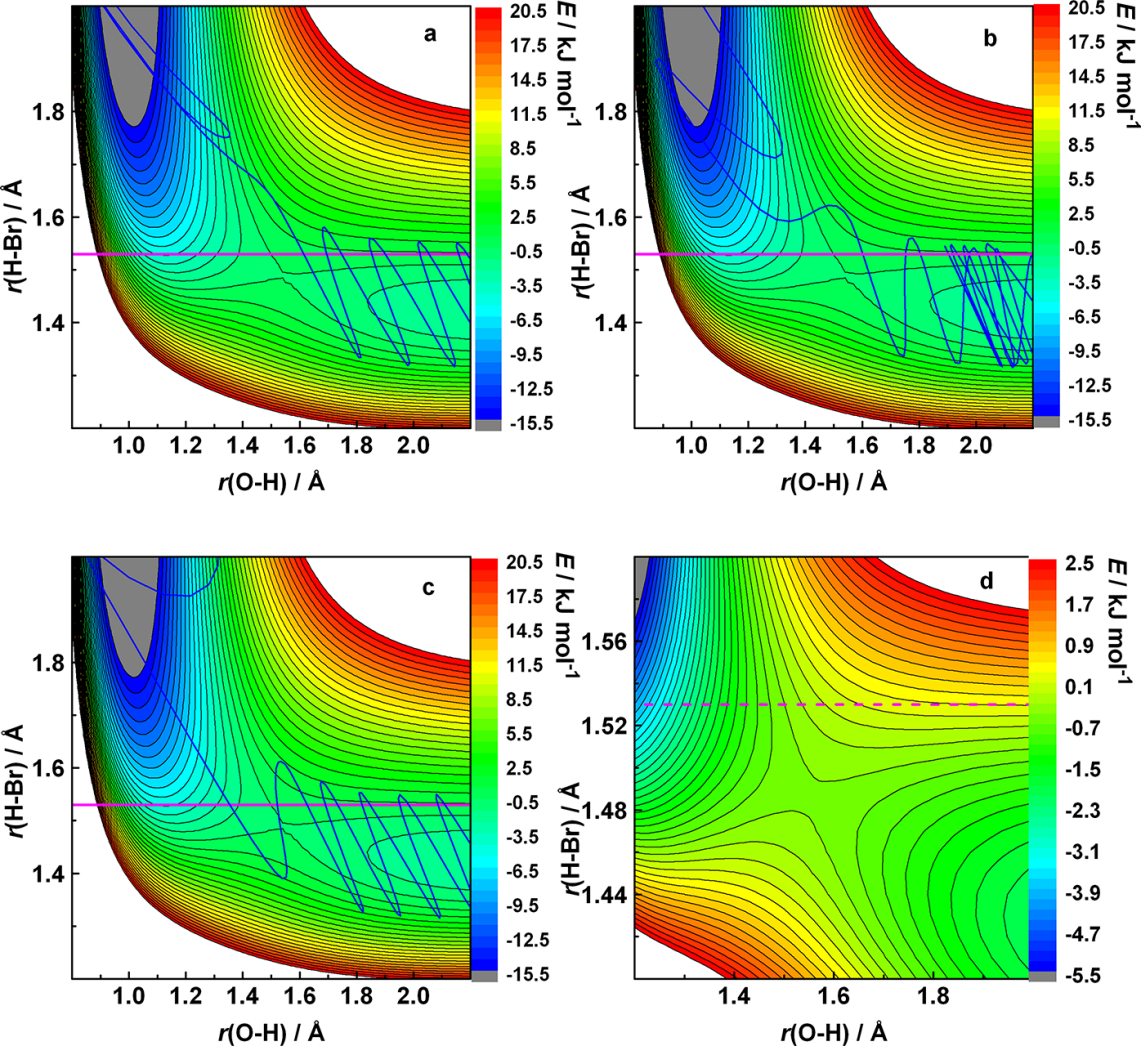
(a–c) Representative trajectories for the HO + HBr (ν = 0) reaction, together with the contour representation of the potential energy surface plotted as a function of the lengths of the forming and breaking bonds. The rest of the geometrical parameters are set to their value at the saddle point of the PES. The collision energy is 0.05 kJ/mol. The contour lines are drawn in 1 kJ/mol steps. The magenta horizontal line is a guide to the eye near the outer turning point of HBr vibration at *v* = 0. Note that the other coordinates also change during the course of the reaction; thus, the section of the PES along the *r*(O–H)–*r*(H–Br) plane in reality also varies slightly between successive time steps. Panel (d) is a magnified view of the same cut of the PES, with energy spacing 0.2 kJ/mol.

As described in the Introduction, for several typical reactions taking place on potential surfaces with a positive barrier, capture-type excitation functions were observed at low collision energies when the breaking bond was vibrationally sufficiently highly excited. The phenomenon was traced back to the behavior of the reacting system near the corner region of the PES. Figure 4 is intended to show that reaction R2 displays the same qualitative behavior (the dynamics of reaction R1 are very similar). Representative reactive trajectories for reaction R2 are projected on the *r*(O–H)–*r*(H–Br) plane, together with the contour plots of the cut of the multidimensional PES along the same plane, with all other coordinates being fixed at the saddle point values. At relatively large reactant separation, where the interaction is weak, the trajectories oscillate between the equipotential lines corresponding to the zero-point energy of the HBr vibration. At large O–H distances, the equipotentials are parallel to the horizontal axis, which reflects that the interaction is small, thus the internal potential of HBr does not change.

Accordingly, the amplitude and the classical action of the H–Br vibration remain constant during the approach of the reactants. At relatively small reactant separation (at around *r*(O–H) = 2.5 Å), the equipotentials at energies corresponding to the zero-point vibrational energy of HBr and above start to turn away from the horizontal axis when *r*(O–H) decreases. The trajectories near the outer turning points of the H–Br oscillation keep touching the same lines, which means that the vibration remains adiabatic. Since the equipotentials are bent, when the trajectory arrives close to them, a force component arises parallel to the forming bond, which slightly accelerates the approach of the reactants. This is reflected in the growing horizontal distance between the successive OTPs. This is a manifestation of the same attraction that was observed for other reactions when the breaking bond was vibrationally highly excited (see Figure S1). When the trajectory reaches the equipotential corresponding to the available energy in the (so far adiabatic) vibration at a point where its curvature is large, then, due to the force component parallel to the *r*(OH) axis, the trajectory is reflected into the product valley (see Figure 4a and b). The motion hardly depends on the presence of the barrier, because it is low. As a result, in many cases the potential barrier is crossed “in the wrong direction”, for example, perpendicular to the minimum energy path (Figure 4c), as if the trajectory “were dropped from above” on the saddle point region. What is remarkable is that in reactions R1 and R2, the phenomenon appears at the lowest physically possible vibrational energy content of the reactant, i.e., the zero-point energy.

The trajectory in Figure 4b allows a glimpse at the role of the pre-reaction potential well. In this specific collision, the trajectory spent a long time in the well. Temporarily, the amplitude of the vibration of HBr increased, due to the coupling with the relative translation. This coupling was induced by the motion along the degrees of freedom not visible in the projection shown in the figure, such as the rotation of the reactants. The energy gained in this coupling was lost by the relative translation, and the trajectory was trapped for about 15 H–Br vibrational periods, because the remaining translational energy was not enough to get out of the well either across the barrier or back to reactants, until the coupling with bending channeled some energy back into this mode.

## Discussion

### Role of the Shape of the PES for Reactions with Extreme Reactivity Enhancement by Vibrational Excitation

**Figure 5:**
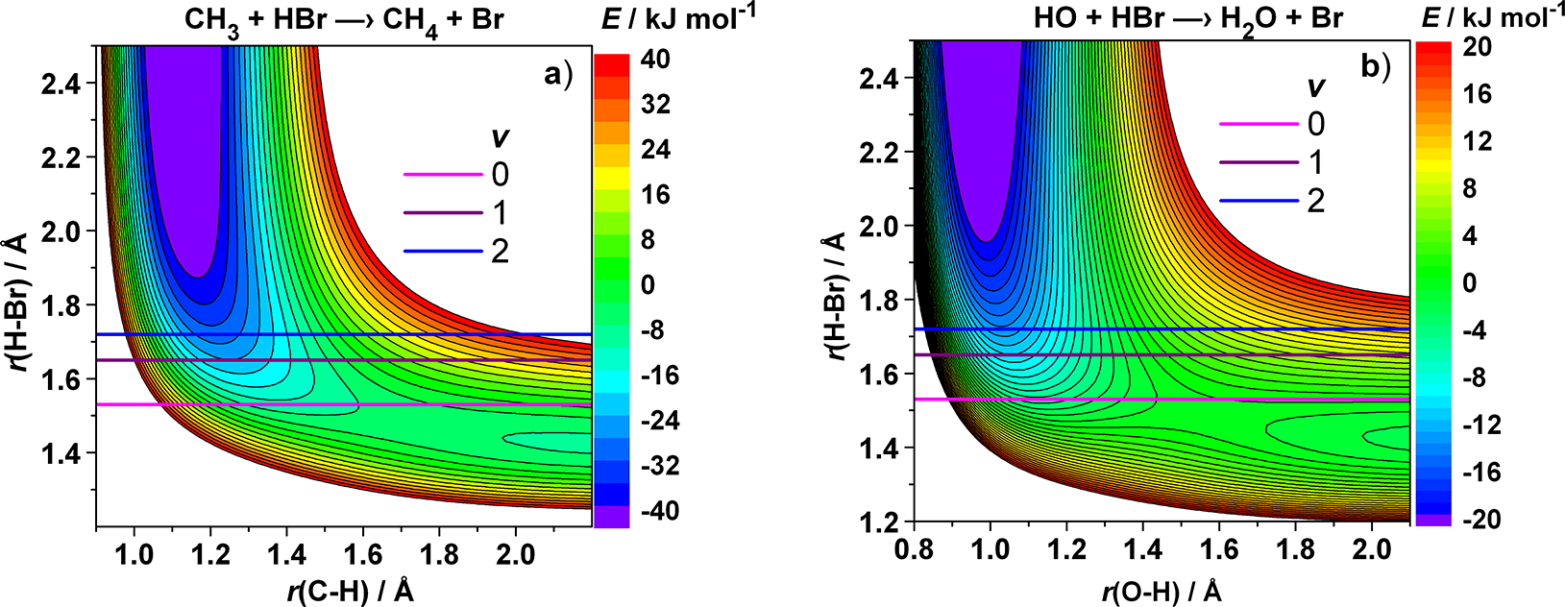
Sections of the potential energy surfaces as functions of the lengths of the forming and breaking bonds for the (a) CH_3_ + HBr and (b) HO + HBr reactions. The colored horizontal lines represent the bond length of the breaking bond at the vibrational outer turning point of HBr at various vibrational quantum numbers. In each panel, the lowest line corresponds to the ground state of HBr, followed by ν = 1, 2. Energies in kJ/mol.

**Figure 6:**
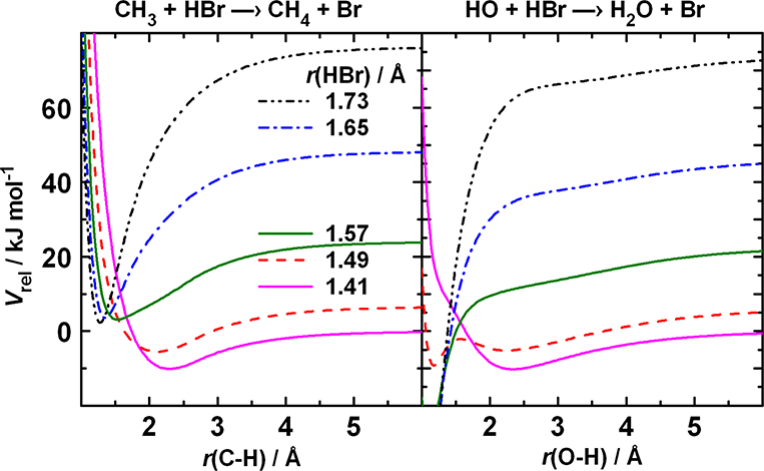
Cuts of the potential energy surfaces of reactions R1 and R2 along the horizontal lines in Figure 5: the potential energy as a function of the length of the forming C–H (left panel) and O–H (right panel) bond at fixed values of the length of the breaking H–Br bond. The top three lines correspond to the outer turning point of the (from top downward) *v* = 2, 1, 0 vibrational states of HBr.

The trajectory calculations indicate that in reactions R1 and R2, the trajectories leading to reaction follow a path similar to what was seen for some other reactions when the vibration of the breaking bond was highly excited. The earlier observations of the appearance of capture-type excitation functions include some highly endothermic hydrogen-abstraction reactions (H + H_2_O, H + HF)^35−39^ with reactant vibrational quantum number above 3, and a close to thermoneutral reaction (H + HCl).^40^ For the latter, the barrier is comparable in height to a vibrational quantum, and the extreme reactivity can be seen already at ν(HCl) = 2. Reactions R1 and R2 are exothermic, and the reactivity enhancement seems to arise already when the reactant HBr is in the vibrational ground state. While the location of the barrier is distinctly different for the highly endothermic, almost thermoneutral, and exothermic reactions, the condition for high reactivity seems to be common for all cases: The amplitude of the vibration of the breaking bond is large enough to ensure that the outer turning point is displaced far from the MEP, to the region of configuration space where the interaction of the reactants is attractive. The attraction is manifested in the shape of the contour lines of the potential energy plotted against the lengths of the forming and breaking bonds: The equipotentials turn away from the horizontal axis when followed right to left (see Figures 4, 5, and S3). Another manifestation of the attraction critical for reactivity enhancement is that the cuts of the PES as functions of the length of the forming bond plotted at fixed values of the length of the extended breaking bond are monotonously attractive (Figure 6). Every time the breaking bond vibration approaches the OTP, a force component arises that is parallel to the direction of approach of the reactants and pulls the reactants toward each other. For early barrier reactions, this is not typical behavior. When the barrier is relatively high and is located at the same early position as for reactions R1 and R2, the topography of a saddle requires that the equipotentials turn toward the horizontal axis, similarly to the three lowest contour lines above the barrier in Figure 4d. The force component arising from the curvature of the contour lines is repulsive and slows down the approach of the reactants. This force component is one of the factors that make vibrational energy less efficient in promoting early barrier reactions than translational energy, as it is familiar from Polanyi’s rules.

Figure 4d and Figure 5 show that for reactions R1 and R2 characterized by a submerged barrier, the repulsive contour lines (those that turn downward when traced from right to left in the figures) are at very low energies, below the reactant level. The reason for this is that the barrier is low even with respect to the well of the prereaction complex. This feature of the PES of reactions R1 and R2 (and very probably many other reactions with submerged, early potential barriers) are exceptional in the sense that the enhancement of the reactivity by vibrational excitation in fact occurs already when the reactants are in the vibrational ground state. Figure 5 shows that the vibrational energy of the vibrationally unexcited HBr bond is so large that for both reactions, on the cut of the PES along the *r*(X–H)–*r*(H–Br) plane (X = C or O), the contour line corresponding to it turns upward with decreasing *r*(X–H). In Figure 6, the cut of the PESs for both reactions are plotted as a function of the length of the forming bond at fixed values of the breaking bond. When the H–Br bond is long, the potential curves monotonously decrease when the length of the forming bond shortens toward its equilibrium distance. Thus, the attraction arising when the zero-point vibration is at the outer turning point effectively supplements the weak nonbonding attraction between the reactants with equilibrium bond lengths. This is the reason the excitation functions for these reactions diverge so quickly when the reactant molecule is vibrationally unexcited. It is reasonable to consider the attraction arising when the breaking bond is significantly stretched as originating from the dynamics of the reacting system, compared with the “static” van der Waals-type interaction that brings together the components of the pre-reaction complex. We shall refer to the attraction due to the large amplitude of vibration as “vibrationally induced”. Since this kind of attraction is strong already in the vibrational ground state of the HBr reactant, it is not surprising that when the vibrational energy available for the reactant is increased above the ground-state level by one or two quanta, the cross sections do not increase as much as one might expect. The vibrational excitation certainly increases the amplitude of the HBr bond length oscillation above that of the vibrational ground state. However, the reactivity enhancement caused by the zero-point oscillation is so large that increasing the amplitude further will not induce as large a change as vibrational excitation of the reactant would in the absence of the extra, dynamically induced attraction.

These arguments seem to provide (one of) the reasons why the reactivity enhancement by vibrational excitation of HBr is larger for reaction R1 than for R2. Figure 5 shows that for reaction R2 the contour lines turn away from the horizontal axis starting from about the HBr bond length corresponding to the OTP of the zero-point vibration, while for reaction R1, the effect occurs at significantly lower energy. This suggests that for the reaction of HO with HBr, the zero-point vibrational amplitude is at the borderline where the vibrationally induced attraction arises, while for the reaction of CH_3_, the zero-point amplitude is well above the limit. One can expect that for reaction R1 the zero-point vibration induces a reactivity enhancement that approaches an upper limit, and by exciting the HBr vibration, the reactivity cannot increase very much. In reaction R2, the reactivity enhancement generated by the zero-point vibration is still not as close to the upper limit, and there is room for further enhancement when a vibrational quantum is added. When the vibrational excitation is increased further, the reactivity will not increase significantly for either reaction, because the vibrationally induced attraction almost fully operates already at *v* = 1.

### Dynamics at Small Vibrational Amplitudes

**Figure 7:**
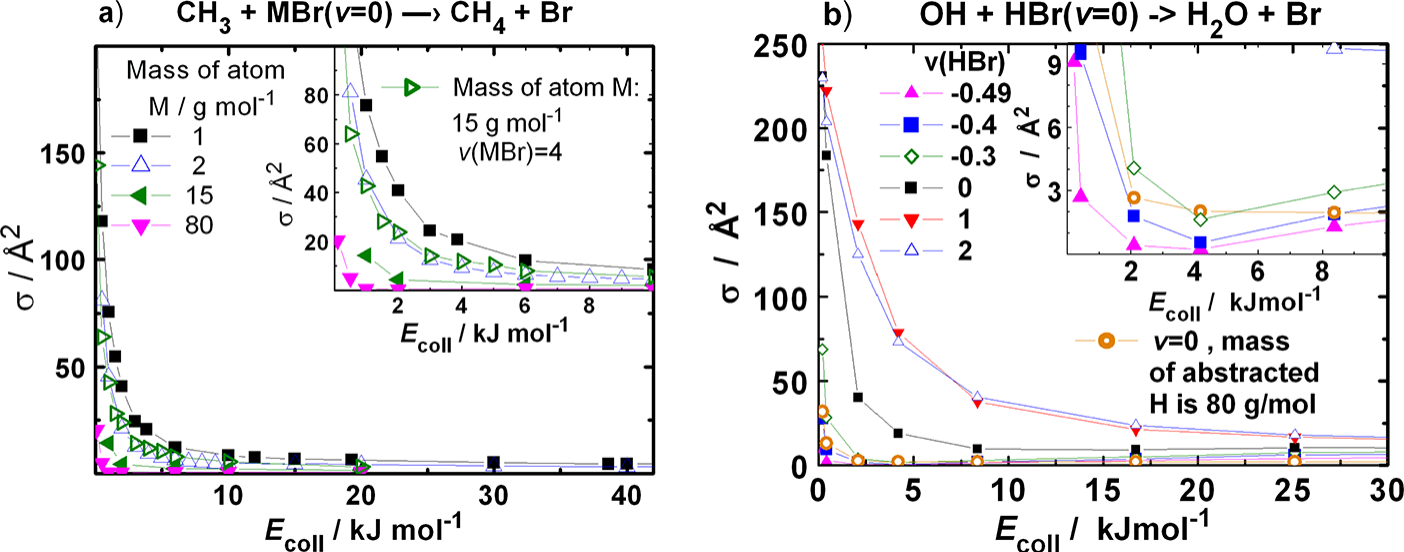
Excitation functions for (a) reaction R1 with the masses of the transferred H atom set to 1, 2, 15, and 80 g/mol (left panel) with ν(HBr) = 0 and with ν(HBr) = 4 for mass 15 g/mol; (b) for reaction R2 with vibrational quantum numbers −0.49, −0.4, 0, 1, and 2 with protium mass as well as with mass for the H atom set to 80 g/mol, combined with ν(HBr) = 0.

In QCT simulations, by directly controlling the vibrational amplitude of HBr, one can test whether really the large amplitude of the ground-state HBr vibration is responsible for the large reactivity. One possibility is that one increases the mass of the H atom that can be abstracted from the HBr molecule. Experimentally, this can be done by deuterium substitution, but since the amplitude decreases roughly as the 1/4th power of the ratio of the mass of protium to that of the heavy isotope, the observable consequences are not spectacular. In simulations of collision dynamics in QCT calculations, one has more freedom and can increase the mass arbitrarily. Another way to reduce the amplitude of the HBr vibration that is not available in experiments is that one reduces the vibrational action below the 1/2 *h*ν semiclassical prescription. We used both methods. In one set of simulations, we increased the mass of the reactive H atom to 2, 15, and 80 g/mol (the latter two values match those of the group/atom between which the H atom is transferred). In the other set of calculations, we reduced the vibrational quantum number of HBr from 0 to −0.4 and −0.49, i.e., decreased the vibrational energy, respectively, to 20% and 2% of the semiclassical zero-point energy. For some cases, we combined the two methods. Figure 7 shows the change of the excitation functions due to the variation of the mass of the abstracted H atom for reaction R1 and the influence of the initial vibrational quantum number of HBr for reaction R2. The reactivity drastically drops both when one increases the mass of the H atom and when the vibration is frozen.

To test the whether the larger inertia of the reactant that accompanies the increase of the mass of the H atom of HBr can influence the reactivity beyond reducing the vibrational amplitude, we calculated the excitation functions with combined variation of mass and vibrational quantum number. For reaction R1, increasing the mass of the H atom to 15 g/mol at ν_HBr_ = 0 reduces the cross section at *E*_coll_ = 2 kJ/mol from 40.9 to 4.3 Å^2^ (see Figure 7). The reactivity, however, can be recovered by vibrational excitation of HBr by increasing the vibrational quantum number to 4 (the respective cross section becomes 25.3 Å^2^). This proves that the mass change predominantly affects the reactivity via reducing the vibrational amplitude, and the lower velocity of approach has a small, probably negligible effect. The mass effect is excluded when the vibrational amplitude is decreased by diminishing the respective quantum number. In Figure 7b, one can see that the reaction can be frozen this way too. In both sets of calculations, the excitation functions turn upward at very low collision energies even when the mass of the transferred H atom is set to very large values or when the vibrational “quantum number” is made very small. This remaining capture-type reactivity shows the effect of the “static” attraction, which is rather small when it is not assisted by the vibrationally induced attraction.

The calculations with artificially reduced vibrational amplitude indicate that the lack of vibrational energy inhibits the reaction. In reality, complete quenching of reactant vibration is not possible, because it is quantized and the lowest energy level, the zero-point energy, is finite. In reactions R1 and R2 the corresponding amplitude is large enough to allow the system to visit the region of the PES where attraction arises between the reactants. This means that the large reactivity in these systems is made possible by the existence of zero-point energy, which is the manifestation of an interesting quantum effect.

**Figure 8:**
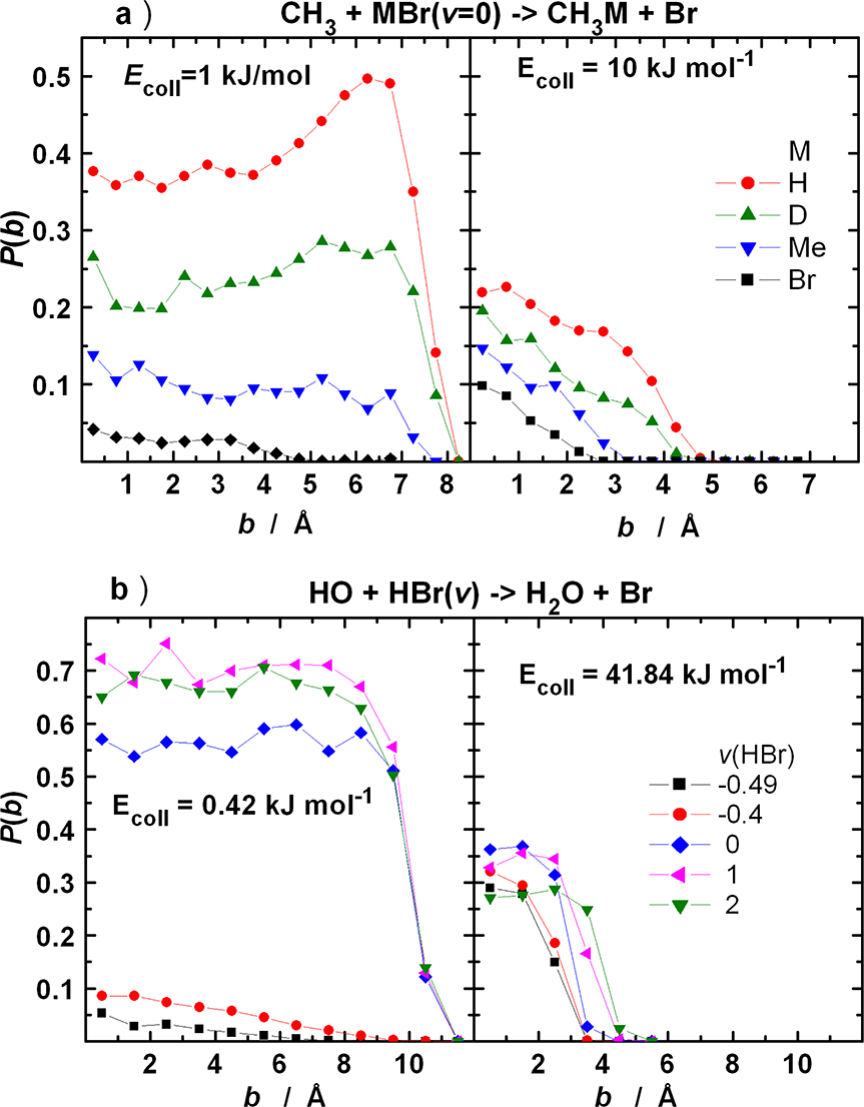
Opacity functions of (a) reaction R1with different masses of the H atom (denoted as M) of the HBr molecule and (b) reaction R2 with various vibrational quantum numbers of HBr. All other degrees of freedom are in the ground state.

It is instructive to look at the effect of changing the mass of the transferred atom or the vibrational quantum number of the HBr reactant on the opacity functions underlying the reaction cross sections. Figure 8a shows that when the mass of the reactive H atom increases, the opacity functions shrink both in height and in width, and their character changes: the large-impact-parameter range becomes completely nonreactive when the mass of the atom to be transferred is large. At low collision energy (left panels), the shape of the opacity functions for reaction R1 reflects capture-type behavior when the mass of the reactive H atom is small (1 or 2 g/mol): the reaction probabilities are very large up to large impact parameters, with a sudden falloff. In contrast, at large masses the probabilities are drastically smaller at small impact parameters and decrease quickly and monotonously with increasing impact parameter. At larger collision energies, where the reactant’s attraction has a smaller influence on the dynamics, the opacity functions decrease roughly linearly with increasing impact parameter, and no qualitative difference can be seen between the small and large masses of the transferred atom. In Figure 8b, the effect of the variation of *v*(HBr) is displayed. Reduction of the reactant’s vibrational quantum number below 0 induces a qualitative change of the opacity functions, similarly to the increase of the mass of the transferred atom. Remarkably, at low collision energy the difference of the reactivity in reaction R2 is much larger when *v*(HBr) increases from −0.4 to 0 than when it changes from 0 to 1 or 1 to 2, especially when considering that in the former case the increase of the vibrational energy is less than one-half of that in the latter two cases. When *v*(HBr) changes from −0.49 to 0 and −0.4 to 0, the enhancement of the reaction probability at, for example, *b* = 2 Å is roughly a factor of 10 and 5, respectively. In contrast, the probability increases approximately by only a factor of 1.15 when *v*(HBr) grows from 0 to 1, and the reactivity enhancement is negligible when one more vibrational quantum becomes available. These trends show that the vibrational ground state of HBr is close to some “large vibrational amplitude limit” for reaction R2 in the capture regime. As discussed in connection with Figures 5 and 6, this is connected to the shape of the potential energy surface: At small vibrational amplitude, the OTP of the vibration remains close to the minimum energy path, and the system cannot reach the region where the vibrationally induced attraction is effective (see Figures 4d and 5). Under such conditions, the “static” attraction does bring together the reactants, but it is not strong enough to effectively guide them through the barrier. Once the vibrational amplitude of HBr is large enough to allow the reactants to visit the region of the PES where the vibrationally induced attraction arises, significant reactivity enhancement can be seen. Figure 8b shows from another perspective that for reaction R2 this happens somewhere close to *v*(HBr) = 0, and the change is quite sudden.

It is worth noting that at large collision energies, where the attraction between the reactants does not affect the reactivity as much as at low collision energies, the reaction probabilities for reaction R2 are not very sensitive to the reactant’s vibrational quantum number. This agrees with the expectation that the translational energy can be utilized for climbing the barrier of the PES, even when the vibrational amplitude is small. The large vibrational amplitude in this regime cannot increase the reactivity effectively, because the ratio of the time scales of vibration and translation becomes less favorable than when the reactants approach each other slowly. As the relative velocity of the reactants increases, fewer and fewer vibrational periods are completed while the reactants are close to each other, and the oscillation of the length of the breaking bond rarely reaches the outer turning point where the vibrationally induced attraction is effective.

### Connection to Non-IRC Dynamics

For the vibrationally induced attraction to operate, the vibrational amplitude needs to be large in comparison with the width of the reactant and product valleys and of the saddle point region. Thus, an intrinsic property of such reacting systems is that they do not closely follow the minimum energy path. There is a group of phenomena known in the literature where the reaction does not visit certain regions of the PES that are close to the MEP, for example, some van der Waals-type wells. These processes are said to be characterized by non-IRC dynamics^53−55^ (IRC, or intrinsic reaction coordinate in this context is equivalent to pathways involving multiple minima and saddle points contiguously connected by the respective minimum energy paths). Non-IRC dynamics is generally considered to take place when the reactants pass a potential barrier, and for dynamical reasons, the trajectories leave the neighborhood of the product side of the MEP. In a sense, the behavior of reactions where reactivity enhancement is caused by vibrationally induced attraction can be considered a kind of non-IRC dynamics. Here, however, the large deviation from the IRC acts *before* the reactants pass the potential barrier, instead of arising after that. As in reactions R1 and R2 the barrier region is often crossed far from the saddle point itself, there is a large chance of post-transition state non-IRC dynamics in the conventional sense. This can be manifested in the product energy distribution among the translational, vibrational, and rotational degrees of freedom, but can have chemical consequences such as roaming. In fact, roaming was found to take place in the H + HF and H + HCl reactions.^39,40^ We plan to study the possible manifestations of post-transition-state non-IRC dynamics for reactions R1 and R2.

### Virtual Violation of Polanyi’s Rule

At first glance, the enhancement of the reactivity of an early-barrier reaction by vibrational excitation seems to violate one of Polanyi’s rules. The latter states that when the potential barrier is shifted to the reactant valley, if one provides the same energy in the form of translation or vibration, the former is more favorable in promoting the reaction. The reason is that the collision energy is deposited in the degree of freedom that is directed along the uphill valley toward the crest of the barrier, so it can be fully utilized by the reactive system to surmount the potential barrier. Vibration, in contrast, corresponds to motion in the perpendicular direction, and is *de facto* useless in climbing the barrier. It can be utilized for surmounting the potential barrier only if it is channeled to the right, uphill direction. If the potential barrier is early, the coupling to translation is rather limited, and the vibration remains completely passive. In terms of the potential energy surface, for reactions for which Polanyi’s rules are designed, the early potential barrier is relatively high or at least positive, and the contour lines on the reactant side of the barrier remain repulsive up to large distances from the minimum energy path (in the cuts the contour lines turn toward the forming bond axis). Even for this kind of reaction, the “vibrationally induced” attraction does arise when the amplitude of the vibration of the breaking bond is large. The attraction facilitates the reaction and even induces enormous rate enhancement, such as that observed experimentally and explained by theory for the reaction of vibrationally highly excited water with H atoms.^36−40^ In the previous sections we have seen that the same factors determine the reactivity when the potential barrier to reaction is early and is submerged so that it forms a reef next to a shallow prereaction potential well. Vibrational excitation—in fact, already when it is relatively low—in such cases *does* promote the reaction, which might sound like a violation of Polanyi’s rule. However, the rule also includes a comparison with the efficiency of collisional energy. The translational energy in the cases of vibrationally induced attraction, instead of promoting the reaction, is not favorable; instead, the reactivity decreases with increasing collision energy. Thus, the question, “in which mode should one provide the same energy to achieve larger reactivity?” is meaningless. Polanyi’s rules apply to systems which more-or-less follow the minimum energy path. This is not the case when the vibrational amplitude is large, so one should not expect the rules to apply to this kind of reactivity enhancement. It remains to be seen whether the extension to Polanyi’s rules, the sudden vector projection model of Guo and co-workers^56,57^ can explain this phenomenon.

## Summary

The effect of vibrational excitation on the reactivity of systems whose potential energy surfaces are characterized by a potential well corresponding to a prereaction complex and a submerged barrier acting as a reef in the formed “lagoon” were studied. For reactions of CH_3_ and HO radicals with HBr, the QCT calculations revealed unexpectedly large reactivity: the reaction cross sections diverge swiftly when the collision energy is reduced. In addition, both reactions are accelerated when the HBr reactant is vibrationally excited. The capture-type excitation functions observed for this reaction repeat what has been seen for the HO + HBr reaction. The large reactivity has been traced back to a static component determined by the shape of the potential energy surface and a dynamical factor originating in the dynamics of the motion of atoms. The static factor is that the potential energy decreases on the path leading into the prereaction potential well when the distance between the reactants diminishes. The dynamical factor comes from the bond length oscillation of the breaking H–Br bond, the amplitude of which is large already in the vibrational ground state of HBr. The oscillation generates extra attraction between the reactants, essentially independent of whether there is a barrier to the reaction. The additional attraction arises when the reacting system leaves the close neighborhood of the minimum energy path (MEP). In particular, when the HBr molecule is fully stretched, the trajectories visit the region of the PES where, as a function of the length of the forming bond, the potential is attractive. The larger the vibrational excitation, the larger the attraction in the stretched phase of the reactant vibration is, and the larger the reactivity. The curiosity of the reactions studied here is that the potential energy surface allows large-amplitude vibration even in the vibrational ground state of the reactants. As a consequence, the divergence of the excitation function is enhanced more by “vibrationally enhanced attraction” than by static attraction. In test calculations, the reactant vibrational amplitude was artificially decreased by increasing the mass of the abstracted atom or by reducing the vibrational action below the zero-point value, which resulted in enormous reduction of the reactivity. The excitation functions are capture-type even in this case, which is due to the attractive potential leading into the prereaction potential well, but the magnitude of the residual reactivity is much smaller than what the “vibrationally enhanced attraction” induces. This means that the zero-point energy of the reactant vibration is necessary to make these reactions as fast as it is measurable in experiments. The reactions would be much slower without z.p.e., which is a unique quantum effect.

The conditions inducing the vibrational enhancement of the reactivity of this kind of early barrier reactions are different from those that govern reactions that more closely follow the MEP, for which Polanyi’s rules have been designed. Thus, these reactions are beyond the sphere of applicability of these rules.

## Supporting Information Available

Plots of potential energy surfaces for the H+HF reaction and the vibrational enhancement factors for reactions R1 and R2 (PDF)
