# Supplementary material for: The Role of Zero-Point Vibration and Reactant Attraction in Exothermic Bimolecular Reactions with Submerged Potential Barriers: Theoretical Studies of the R + HBr → RH + Br (R = CH3, HO) Systems
Source: J Phys Chem A. 2021 Sep 20;125(38):8386–96. doi: 10.1021/acs.jpca.1c05839 (PMC8488937; doi:10.1021/acs.jpca.1c05839)
Supplement: Supplementary file 1 — jp1c05839_si_001.pdf [file jp1c05839_si_001.pdf]

Supporting Information to Manuscript

jp-2021-05839t

The role of zero-point vibration and reactant attraction in exothermic bimolecular reactions with submerged potential barriers: theoretical studies of the R + HBr → RH + Br (R = CH_3, HO) systems

Benjámin Csorba[1], Péter Szabó[1], Szabolcs Góger[1] and György Lendvay[1,2]

[1] Institute of Materials and Environmental Chemistry, Research Centre for Natural Sciences, Magyar tudósok krt. 2., H-1117 Budapest, Hungary

[2] Center for Natural Sciences, Faculty of Engineering, University of Pannonia, Egyetem u. 10. Veszprém, 8200 Hungary

Contents

Fig. S1. Plots of potential energy surfaces for the H+HF reaction

Fig. S2. The vibrational enhancement factors for reactions CH_3 + HBr → CH_4 + Br (R1) and HO + HBr → H_2O + Br (R2).

Figure S1. Sections of the 6-SEC[1] potential energy surface of the H + H'F → HH' + F reaction. a) the energy as a function of the location of the attacking H atom with respect to the H' atom located at the origin of the coordinate system. The F atom points downward; the H'–F bond length is 0.917, 1.15 and 1.35 Å on the three panels from left to right. The outer turning point of the vibration at v_{HF} = 4 is 1.324 Å. b) The potential energy of the H–H'–F arrangement according to the 6-SEC PES plotted against the H–H' distance as a function of the H–H distance when the H'–F bond length is fixed at various values. The H–H'–F angle is 104° (the saddle point value). The outer turning point of the vibration at v_{HF} = 4 is 1.324 Å.

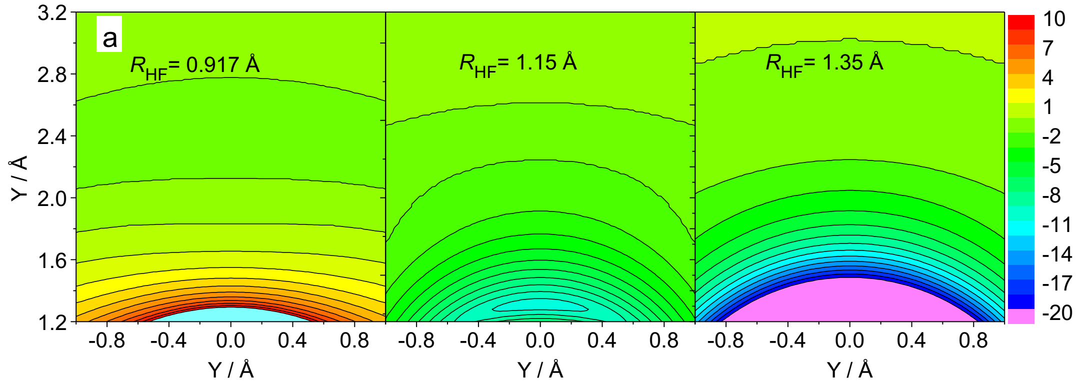

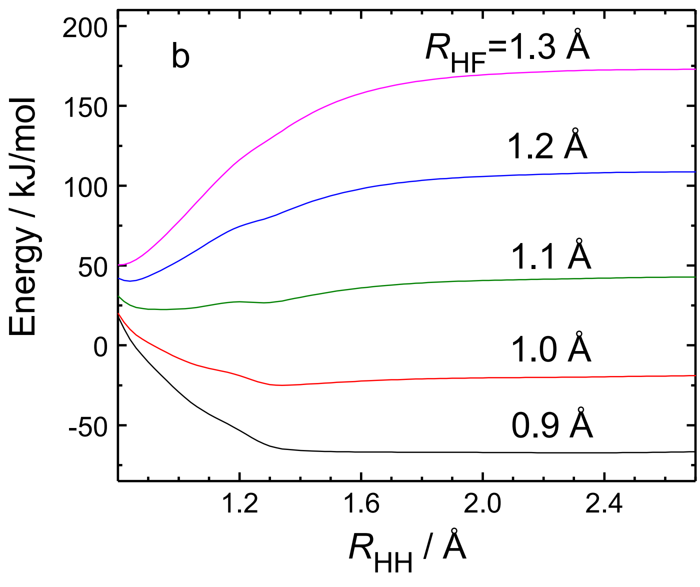

Figure S2. The vibrational enhancement factors for reactions (R1) and (R2): ratio of reaction cross sections for vibrationally excited vs. unexcited HBr.

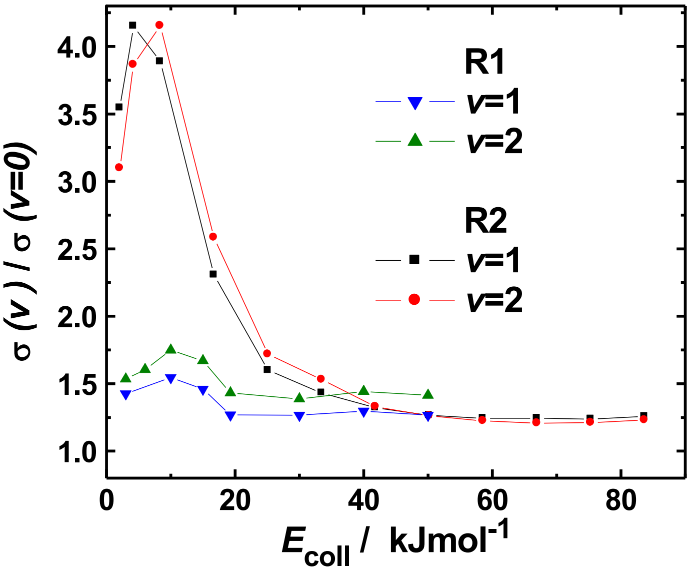

References

[1] Mielke, S. L.; Lynch, G. C.; Truhlar, D. G.; Schwenke D. W., *Chem. Phys. Lett.* **1993**, *213*, 10; Erratum *ibid.* **1994**, *217*,173
